# Self-managed abortion via the internet: Analysis of one year of service delivery data from Women Help Women

**DOI:** 10.12688/gatesopenres.14369.1

**Published:** 2023-02-13

**Authors:** Tara Shochet, Lucía Berro Pizzarossa, Sara Larrea, Jennifer Blum, Kinga Jelinska, Rodica Comendant, Irina Sagaidac

**Affiliations:** 1 Gynuity Health Projects, New York, NY, USA; 2 Women Help Women, Amsterdam, The Netherlands; 3 Independent Researcher, Amstersdam, The Netherlands; 4 Reproductive Health Training Center, Chisinau, Moldova; 5 Nicolae Testemitanu State University of Medicine and Pharmacy, Chisinau, Moldova

**Keywords:** medication abortion, self-managed abortion, telehealth

## Abstract

**Background:** To better comprehend the demand for online medication abortion and to inform service delivery practice, we conducted an analysis of Women Help Women (WHW) service delivery statistics. The primary goals were to understand their user profile, evaluate self-reported outcomes and use of other medical services, and assess the overall experience both with the abortion itself and with the counseling and care provided by WHW.

**Methods: **We retrospectively evaluated user characteristics, abortion outcomes, and acceptability of both the medication abortion and WHW’s services, using consultation data and corresponding evaluation data from a one-year period. For users who did not complete the evaluation form, WHW staff reviewed email correspondences to identify key outcomes.

**Results: **From August 2016-July 2017, 3,307 individuals received abortion pills from WHW. Users were geographically located in thirty countries and correspondence was conducted in seven languages. Most reported their gestational age to be less than eight weeks. Of the 2,295 who took the pills and provided outcome information, almost all (99.1%, n=2275) reported that they were no longer pregnant. The majority (84.1%, n=1576/1875) used symptoms to confirm outcome; one fourth (22.8%, n=428) sought an ultrasound and one sixth (18.0%, n=338) used urine and/or serum testing. One in eight users (12.6%, n=292/2317) reported seeking additional medical care after taking the abortion pills. Most (87.5%, n=1551/1773) reported being satisfied or very satisfied with the abortion.

**Conclusions: **Our study confirms that self-managed abortion is a process that people can do safely and effectively with community support and without medical supervision. In the context of a global backlash against abortion rights, self-managed abortion is an integral part of a spectrum of options for abortion care that must be made available to all.

## Introduction

The COVID-19 pandemic has catalyzed simple, less medicalized models of abortion services—such as telemedicine—and spotlighted the work of many organizations offering support and access to medicines in this way ^1, 2^. This has bolstered the growing global interest in both obtaining medication abortion drugs and self-managing abortions outside of the institutionalized healthcare system ^3, 4^. Increasing evidence demonstrates the high safety and efficacy of online telehealth abortion ^5– 8^; thousands of pregnant individuals have utilized telehealth services to access medication abortion medicines worldwide ^9– 12^.

Preceding telemedicine by many decades, feminist organizations have long organized to provide a more comprehensive source of information and reliable medicines to people self-managing their abortions outside of institutional systems of health care ^13, 14^. Advances in medical technology—namely the medicines misoprostol and mifepristone— coupled with community provision of information and support have drastically increased the safety and effectiveness of self-managed abortion (SMA) over the last 50 years ^12, 15– 17^. Feminist activism for SMA began at the margins of health systems and has developed and diffused de-medicalized practices that have spread globally ^18^. Indeed, building user-friendly systems and empowering communities and individuals with information to become agents of their own health has transformed the reproductive health field and dramatically improved health outcomes in Latin America and the Caribbean (LAC) and other regions ^19^.

Self-managed abortion has been recognized for its potential to contribute to personal agency and reproductive freedom and shift power away from the institutionalized medical system and into the hands of pregnant people. Qualitative research has shown that some individuals seek self-managed abortion due to comparative advantages in terms of privacy, comfort, and convenience while also citing financial and logistical barriers to accessing clinical care ^20– 23^.

The World Health Organization (WHO), The Royal College of Obstetricians and Gynaecologists (RCOG), and The American College of Obstetricians and Gynecologists (ACOG) all recommend the use of telehealth for abortion in the first trimester ^24– 26^. Moreover, in 2022, the World Health Organization (WHO) revised their abortion care guidelines and recognized self-management, as well as accompaniment from trained community health professionals, as evidence-based abortion provision, highlighting their roles in improving access, privacy, and convenience in restricted settings and beyond ^26^. 

Women Help Women (WHW) ( https://womenhelp.org/), an international non-profit organization, forms part of the constellation of actors that work locally and transnationally, enabling SMA access and providing different types of support ^27^. Women Help Women was established in 2014 to expand access to abortion worldwide via the internet and through on-the-ground partnerships with local activist groups. The WHW website provides detailed information about medication abortion, access to skilled counselors who provide information and support in eight languages, and an opportunity to access abortion pills directly. Abortion seekers can complete a consultation form that is reviewed by WHW staff. The pills are mailed if the individual qualifies for medication abortion services and doesn’t have any medical contraindications. If eligibility is uncertain, a clinician does an additional evaluation prior to the package being sent. Users are asked to donate 75 Euros to help maintain the service, but those who cannot afford it are asked to donate what they can. Two weeks after receipt of the package, the user receives an email from WHW with a link to a web-based evaluation survey that assesses abortion outcome and overall experience with the abortion and service. At any time during the process, users may email WHW to ask questions or share concerns; prompt responses are sent in the person’s preferred language.

To better comprehend the demand for online medication abortion and to inform service delivery practice, we conducted an analysis of WHW service delivery statistics. The primary goals were to understand their user profile, evaluate self-reported outcomes and use of other medical services, and assess the overall experience both with the abortion itself and with the counseling and care provided by Women Help Women.

## Methods

### Ethical statement

The study was approved by Allendale IRB, USA; informed consent was waived as all data was de-identified and personal or identifying information was not shared with the research team.

### Study design

Women Help Women and Gynuity Health Projects retrospectively evaluated user characteristics, abortion outcomes, and acceptability of both the medication abortion and WHW’s services, using data from a one-year period. All consultation data from August 1, 2016-July 31, 2017, were extracted from the WHW database, as were corresponding evaluation data.

Consultation data included demographics (such as date of birth, country of residency, and language spoken), questions regarding the pregnancy (including date of last menstrual period and if/how pregnancy confirmation was ascertained), and confirmation that the person no longer wishes to be pregnant. Additional medical questions were also asked, as warranted, in order to ensure patient safety and to determine eligibility for medication abortion. Examples of such questions included asking details about any reported health conditions (date of diagnosis, whether the condition was under control, if a clinician was providing care), and gathering more information about any medications being taken on a routine or current basis. Evaluation data included if/when the abortion pills were taken, information about outcome and how outcome was confirmed, whether or not additional care was sought, and a series of questions on satisfaction. For users who did not complete the evaluation form, WHW staff reviewed email correspondences to identify key outcomes pre-identified by the Gynuity research team. (Gynuity, in collaboration with WHW staff, developed a data collection matrix for the staff to complete, to complement the data available in the standard evaluation form.) These outcomes included whether the pills were taken, if the pregnancy had ended, method of confirming outcome, and additional medical care received.

Data extracted from the online system were exported into excel (version unknown) by the WHW staff and then uploaded into Stata/SE 12.1 (College Station, TX). Findings from the data collection matrix were entered into a separate database and then merged with the primary data prior to analysis. We excluded anyone who completed the consultation but did not receive abortion pills from WHW. Analyses are primarily descriptive, examining frequencies and medians. We also compared subgroups using Fisher’s exact tests.

## Results

**Figure 1.**
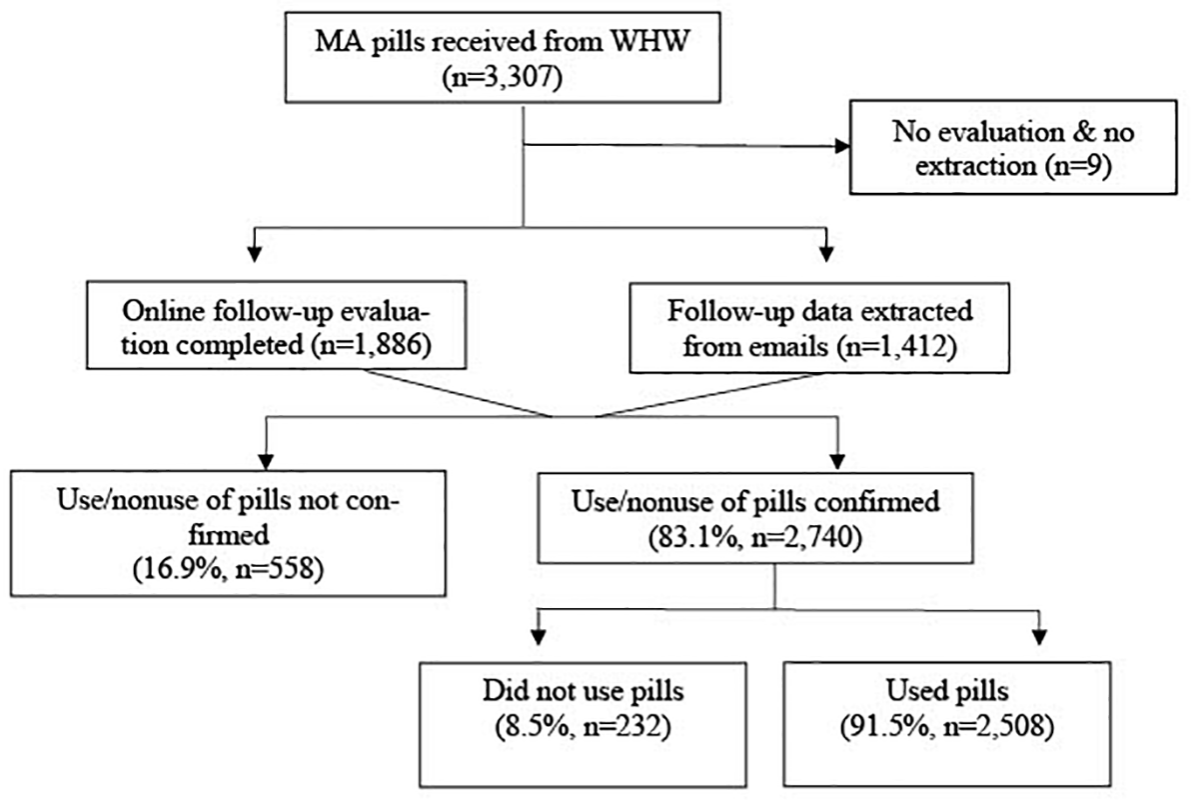
Participant flow of our selected sample.

From August 1, 2016-July 31, 2017, 3,307 individuals received abortion pills from Women Help Women. Figure 1 shows the study flow from pills requested to pills taken. Users were geographically located in 30 countries, with more than half (54.6%) living in South and Central America, about a quarter (22.1%) in Western Europe and the remaining quarter were comprised of residents in Eastern Europe (15.5%), Asia (7.6%), and Africa (0.1%; Table 1). Correspondence between these individuals and WHW staff was conducted in seven languages (data not shown for security purposes). The median age was 27 years and ranged from 14 to 47. Most (88.4%, n=2433) reported their gestational age at time of contact with WHW to be less than eight weeks (≤55 days) since last menstrual period. Most had confirmed their pregnancy status with a urine pregnancy test (82.3%, n=2,687), while 27.9% (n=910) reported getting a serum pregnancy test and 17.9% (584) had had an ultrasound. Nearly a quarter (24.1%; n=787) used more than one method to confirm their pregnancy status. Confirmation method used was associated with geographic location (data not shown for security purposes).

**Table 1.** Demographics and background information for all individuals who completed online consultation and received MA pills from WHW %(n) or median (range).

	Individuals who received MA pills from WHW (n = 3,307)
Region of origin South & Central America Western Europe Eastern Europe Asia Africa	54.6 (1,807) 22.1 (732) 15.5 (513) 7.6 (252) 0.1 (3)
Age:	(n=3,298) 27 (14-47)
Age groups: <20 20–24 25–29 30–34 35–39 40–44 45–49	(n=3,298) 11.5 (378) 29.3 (965) 22.8 (753) 19.5 (642) 12.1 (399) 4.5 (148) 0.4 (13)
GA in weeks at start of consultation, by group: <8 weeks (≤ 55 days) 8 weeks (56–62 days) 9+ weeks (63+)	(n=2,752) 88.4 (2433) 9.4 (258) 2.2 (61)
Method used for pregnancy confirmation: Urine pregnancy test Serum pregnancy test Ultrasound Multiple methods used	(n=3,264) 82.3 (2,687) 27.9 (910) 17.9 (584) 24.1 (787)
Reasons for seeking abortion ^a^ Does not want to have (more) children (yet) Financial issues It will interfere with job /education Too young or too old Issues with partner/family Single (not partnered) Other Prefer not to say	57.1 (1889) 48.6 (1608) 37.6 (1245) 33.8 (1118) 23.3 (769) 17.3 (571) 9.0 (298) 15.9 (526)

^a^ Some individuals provided more than one reason

Reasons for seeking abortion varied, with individuals commonly citing a desire to either avoid or postpone future childbearing (57.1%, n=1889), financial difficulties (48.6%, n=1608), concerns about work or education (37.6%, n=1245), feeling too young or too old (33.8%, n=1118), issues with their partner or family (23.3%, n=769), and/or because they were single (17.3%, n=571).

We were able to obtain final outcome information for 2295 of the 2508 (91.5%) people who reported taking the pills ( Table 2). Almost all (99.1%, n=2275) confirmed that they were no longer pregnant. Twenty(0.9%) reported still being pregnant at time of last follow-up contact, 19 of whom said they had taken the abortion medications. The majority (84.1%, n=1576/1875) used symptoms, including seeing products of conception, no longer feeling pregnant, and/or returned menses to confirm they were no longer pregnant. Almost one fourth (22.8%, n=428) sought an ultrasound for outcome confirmation, and around one sixth (18.0%, n=338) used urine and/or serum testing. As with method of pregnancy confirmation, method of outcome confirmation also varied by country (data not shown for security purposes).

**Table 2.** Self-reporting of outcomes among individuals who confirmed having used abortion pills from WHW % (n).

	n = 2,508
Final outcome No longer pregnant (includes individuals who received additional pills and/or surgical intervention or had miscarriage) Still pregnant, continued pregnancy Final pregnancy status unknown	99.1 (2275/2,295) 0.9 (20/2,295) n=213
Method used to confirm abortion outcome ^a^ Symptoms (seeing products of conception, no longer feeling pregnant, and/or returned menses) Ultrasound Urine pregnancy test Blood pregnancy test	(n=1,875) 84.1 (1576) 22.8 (428) 12.5 (234) 7.0 (131)
Took the pills within 1 week of receipt	92.5 (1,442/1,559)
Received medical care after taking pills	12.6 (292/2317)
Reason for seeking medical care ^a^ Excessive or prolonged bleeding Pain Abnormal vaginal discharge Fever No or minimal bleeding/no expulsion/retained POC/incomplete abortion For confirmation of success Other reason	(n=2317) 5.0 (114) 3.2 (74) 1.0 (23) 0.7 (17) 0.7 (17) 0.6 (13) 1.0 (24)
Treatment received ^a^ Surgical intervention (D&C or vacuum aspiration) Antibiotics Additional misoprostol Blood transfusion Other, not specified	(n=2,317) 4.4 (102) 2.5 (57) 0.8 (19) 0.5 (12) 3.2 (74)

^a^ Some individuals reported more than one method/reason/treatment

Compliance was assessed through documentation of administration of abortion medication as instructed. The vast majority of users with follow-up information reported taking the abortion pills (91.5%, n=2508/2740); most who reported time frame of administration indicated taking them within one week of receipt (92.5%, n=1442/1559).

One in eight users (12.6%, n=292/2317) reported seeking additional medical care after taking the abortion pills. Reasons for seeking additional care included for management of excessive or prolonged bleeding (5%, n=114), pain (3.2%, n=74) and/or other medical indications as shown on Table 2. A small number of users (n=13), sought care just to confirm success. Additional clinical care received included surgical intervention (4.4%, n=102), antibiotics (2.5%, n=57) and/or additional misoprostol (0.8%, n=19). Twelve users who sought care for excessive or prolonged bleeding reported that they received a blood transfusion.

Users were asked to report any side effects experienced after taking the abortion medications ( Table 3). Pain was the most commonly reported side effect (94.5%, n=1675), followed by chills (72.1%, n=1278), diarrhea (69.4%, n=1230), nausea (65.3%, n=1158), vomiting (51.8%, n=919), and fever (48.7%, n=863). Approximately one in five individuals (21.5%) reported the pain to be the highest level possible. Other side effects were considered intolerable for approximately 10–18% of users. Among those with the most severe pain, 88% (n=336/381) were satisfied or highly satisfied with the abortion.

**Table 3.** Self-reporting of side effects among individuals who took MA pills received from WHW and who completed online evaluation form % (n).

	(n = 1,773)
Pain Yes No Proportion of individuals with worse pain *	94.5 (1675) 5.5 (98) 21.5 (381)
Chills Yes No Proportion of individuals with intolerable chills *	72.1 (1278) 27.9 (495) 11.6 (206)
Diarrhea Yes No Proportion of individuals with intolerable diarrhea *	69.4 (1230) 30.6 (543) 13.8 (244)
Nausea Yes No Proportion of individuals with intolerable nausea *	65.3 (1158) 34.7 (615) 13.4 (237)
Vomiting Yes No Proportion of individuals with intolerable vomiting *	51.8 (919) 48.2 (854) 17.5 (310)
Fever Yes No Proportion of individuals with intolerable fever *	48.7 (863) 51.3 (910) 10.0 (178)

* Pain was asked as 1-5, scale where 1 was “no pain” and 5 was “worst pain”. The other side effects were asked as 1-5, scale where 1 was “no side effects” and 5 was “intolerable”

Following the abortion, users were asked to share how they were feeling emotionally ( Table 4). The majority (85.1%, n=1509) reported feeling relieved and/or resolved; two-thirds (66.4%, n=1178) felt comfortable, confident, strong, and/or happy; and one-third (35.3%, n=625) felt sad, guilty, and/or confused. Less than one tenth (8.5%, n= 150) were grieving and/or disappointed. Almost nine in 10 (87.0%) reported being satisfied or very satisfied with the abortion; 6.1% (n=108) were dissatisfied or very dissatisfied. The evaluation form also included questions about the WHW service. Most people (90.4%, n=1611) reported that they were satisfied or very satisfied with the information provided on the WHW website; 5.0% said they were dissatisfied or very dissatisfied. The support provided from the WHW staff was also highly praised; with 90.5% reporting satisfaction or high satisfaction with the individual-level support provided as needed throughout the abortion process via email. Approximately 5% (n=98) were not satisfied with the support provided. Nearly all (98%) would recommend the website to a friend and a similarly high proportion (96.4%) would recommend medication abortion to a friend desiring to end a pregnancy. The suggested donation for the service was considered affordable by three-quarters (74.2%) of users. The majority of users (72.9%) learned about WHW through an internet search. Others heard of the service from friends, other organizations, social media, and/or the news.

**Table 4.** Self reports of acceptability of method and service among individuals who completed evaluation form % (n).

How individual feels about abortion ^a^ Relieved/resolved Comfortable/confident/strong/happy Sad/guilty/confused Grieving/Disappointed Other	(n=1773) 85.1 (1509) 66.4 (1178) 35.3 (625) 8.5 (150) 7.4 (131)
Satisfaction with the abortion, Satisfied or very satisfied (4 or 5): Dissatisfied or very dissatisfied (1 or 2):	(n=1783) 87.0 (1551) 6.1 (108)
Satisfaction with website information Satisfied or very satisfied Dissatisfied or very dissatisfied	(n=1783) 90.4 (1611) 5.0 (89)
Satisfaction with online support provided Satisfied or very satisfied Dissatisfied or very dissatisfied	(n=1783) 90.5 (1613) 5.5 (98)
Would recommend WHW website to a friend	98.0 (1780/1816)
Would recommend medication abortion to a friend who needs an abortion	96.4 (1750/1816)
Suggested donation was affordable	74.2 (1378/1858)
How found out about WHW Internet search Friends From another organization Social media like Facebook, twitter, etc. News	(n=1858) 72.9 (1354) 17.2 (319) 5.4 (101) 2.6 (48) 1.9 (36)

Satisfaction scales have 1-5 range, where 1 is “Very dissatisfied” and 5 is “Very satisfied” ^a^ Some individuals reported more than one feeling

## Discussion

Our analysis details the spectrum of users and their utilization of WHW’s medication abortion service. The vast majority had successful abortions, and most were highly favorable of their experiences and the organization. Almost all the individuals considered in this study would recommend self-managed abortion to a friend, signaling that self-managed abortion with medicines is not just a measure of last resort but a legitimate and appreciated care model that many people find works better for them for a myriad of different reasons ^8, 28– 38^.

The reported prevalence of serious adverse events was low, confirming previous findings from other telehealth abortion services ^5, 6, 8^. The email and text support from WHW that is available throughout the process allows users to check in with any concerns and receive reassurance when no additional care is warranted. Further, the prevalence of adverse events needs to be read in light of the possible outcomes for people forced to resort to more invasive and dangerous methods or carry an unsupported pregnancy to term. More than 70% of the people using this service live in countries where abortion is severely restricted or practically inaccessible.

Our findings also contribute to the existing literature on post-abortion feelings that reports that an overwhelming majority of abortion seekers—95% in a study conducted in the US—felt that termination was the right decision for them and that those feelings of relief predominated five years out ^39, 40^.

This is a retrospective chart review, and therefore we were not able to tailor specific questions or to follow up when additional information was desired. There is also a fair amount of missing data. In addition, all data are self-reported; there were no confirmations of gestational age or outcome. Given the stigma and clandestine nature of abortion in many locations, we believe that this analysis includes a representative sample of service users.

For decades, the work of feminist networks and organizations—like WHW—has shown that self-managed abortion is a safe and effective option for pregnant people. A recent study demonstrated that for pregnancies less than nine weeks duration, abortion completion following self-managed medication use is not inferior to abortion completion following clinic-managed care ^12^. As governments are moving towards less medicalized models of access, our study underlines the importance of supporting people worldwide in safely and effectively self-managing their abortions ^27^.

## Conclusions

Our study confirms that self-managed abortion is a process that people can do safely and effectively with community support and without medical supervision. We show that high quality abortions—safe, effective, supported—are taking place outside of institutional systems of medical care. Thus, in line with this evidence and the most recent technical and human rights standards, governments must fully decriminalize the practice and provide a supportive enabling environment for self-managed abortion ^41^. In the context of a global backlash against abortion rights, self-managed abortion is an integral part of a spectrum of options for abortion care that must be made available to all.

## Data Availability

The data that support the findings of this study was made available by Women Help Women and is subject to security restrictions. The data are not publicly available due to containing information that could compromise the privacy of the service users and counselors and the confidentiality of the service. Data will be made available by the corresponding author on a per-request basis after gathering the permission of Women Help Women. All identifiers (including country and language) will be removed.
