# Supplementary material for: Self-managed abortion via the internet: Analysis of one year of service delivery data from Women Help Women
Source: Gates Open Res. 2023 Feb 13;7:41. [Version 1] doi: 10.12688/gatesopenres.14369.1 (PMC10130357; doi:10.12688/gatesopenres.14369.1)
Supplement: Peer review history

# Peer review: Reviewer response for version 1

**DOI:** 10.21956/gatesopenres.15680.r32977

**Published:** 2023-04-25

**Authors:** Flavia B. Pilecco

**Affiliations:** 1 Department of Preventive and Social Medicine, Universidade Federal de Minas Gerais, Belo Horizonte, Brazil

This study is highly relevant, particularly in the context of the COVID-19 pandemic. Nevertheless, the utilization of secondary data has resulted in a significant loss of subjects, a matter of considerable concern. To address this issue, conducting a sensitivity analysis to determine whether the characteristics of the missing subjects, such as age, region of residence, and gestational age, are comparable to those of the remaining ones would be beneficial. Additionally, the ethical issues arising from the study could be further developed to provide a more comprehensive understanding of the implications of the research. Although the authors assert that the data has been anonymized, there are questions surrounding the methodology employed in handling the correspondence between individuals who received pills and the WHW, which may have compromised confidentiality. Finally, addressing the issue of abortion without testing could strengthen the discussion. During the COVID-19 pandemic, protocols of abortion with no prior test have been proposed based on the successful experiences of NGOs. The current study reveals that the majority of women did not need a post-abortion test to confirm the termination of pregnancy. This finding, in addition to reinforcing the confidentiality and security of the subject, implies a reduction in costs and in the need for qualified personnel, which is important in health emergency scenarios.

Is the work clearly and accurately presented and does it cite the current literature?

Yes

If applicable, is the statistical analysis and its interpretation appropriate?

Yes

Are all the source data underlying the results available to ensure full reproducibility?

Yes

Is the study design appropriate and is the work technically sound?

Partly

Are the conclusions drawn adequately supported by the results?

Yes

Are sufficient details of methods and analysis provided to allow replication by others?

Yes

Reviewer Expertise:

Epidemiology; sexual and reproductive rights; gender

I confirm that I have read this submission and believe that I have an appropriate level of expertise to confirm that it is of an acceptable scientific standard.

# Peer review: Reviewer response for version 1

**DOI:** 10.21956/gatesopenres.15680.r32927

**Published:** 2023-03-17

**Authors:** Beatriz Galli

**Affiliations:** 1 Senior Policy and Advocacy Consultant Ipas, Chapel Hill, NC, USA

This article would benefit if the Conclusions also address the fact that the majority of abortion seekers are from regions with restrictive abortion laws as per statistics indicated in the results section. This data can be useful to support the argument for abortion law decriminalization by states but also the need to ensure national regulatory policies that allow women to self-manage their abortions considering the safety and efficacy of the WHW abortion care model as data indicated. The decriminalization of abortion does not always reflect in enabling environments for women to self-manage their abortions despite WHO international human rights standards and scientific based recommendations.

Is the work clearly and accurately presented and does it cite the current literature?

Yes

If applicable, is the statistical analysis and its interpretation appropriate?

Yes

Are all the source data underlying the results available to ensure full reproducibility?

Yes

Is the study design appropriate and is the work technically sound?

Yes

Are the conclusions drawn adequately supported by the results?

Partly

Are sufficient details of methods and analysis provided to allow replication by others?

Yes

Reviewer Expertise:

international human rights law and public health

I confirm that I have read this submission and believe that I have an appropriate level of expertise to confirm that it is of an acceptable scientific standard.
